# A multi-centre randomised controlled study of pre-IVF outpatient hysteroscopy in women with recurrent IVF implantation failure: Trial of Outpatient Hysteroscopy - [TROPHY] in IVF

**DOI:** 10.1186/1742-4755-6-20

**Published:** 2009-12-03

**Authors:** Tarek El-Toukhy, Rudi Campo, Sesh Kamal Sunkara, Yacoub Khalaf, Arri Coomarasamy

**Affiliations:** 1 Assisted Conception Unit, Guy's and St Thomas' Foundation Trust, London, SE1 9RT, UK; 2 Leuven Institute for Fertility and Embryology, Tiensevest, Leuven, 168 3000, Belgium; 3 Academic Department, Birmingham Women's Hospital, Birmingham, B15 2TG, UK

## Abstract

**Background:**

The success rate of IVF treatment is low. A recent systematic review and meta-analysis found that the outcome of IVF treatment could be improved in patients who have experienced recurrent implantation failure if an outpatient hysteroscopy (OH) is performed before starting the new treatment cycle. However, the trials were of variable quality, leading to a call for a large and high-quality randomised trial. This protocol describes a multi-centre randomised controlled trial to test the hypothesis that performing an OH prior to starting an IVF cycle improves the live birth rate of the subsequent IVF cycle in women who have experienced two to four failed IVF cycles.

**Methods and design:**

Eligible and consenting women will be randomised to either OH or no OH using an internet based trial management programme that ensures allocation concealment and employs minimisation for important stratification variables including age, body mass index, basal follicle stimulating hormone level and number of previous failed IVF cycles. The primary outcome is live birth rate per IVF cycle started. Other outcomes include implantation, clinical pregnancy and miscarriage rates.

The sample size for this study has been estimated as 758 participants with 379 participants in each arm. Interim analysis will be conducted by an independent Data Monitoring Committee (DMC), and final analysis will be by intention to treat. A favourable ethical opinion has been obtained (REC reference: 09/H0804/32).

**Trail Registration:**

The trial has been assigned the following ISRCTN number: ISRCTN35859078

## Background

Only a third of in vitro fertilization (IVF) cycles started end in a pregnancy and one fourth result in a live birth [1,2]. Recurrent IVF implantation failure (two or more failed IVF cycles) is a very distressing experience to patients [3] and increases the financial burden on the couple or service provider. The aetiology of recurrent IVF implantation failure can be broadly attributed to embryonic or uterine factors, but remains unexplained in most cases [4]. A number of interventions have been proposed to improve IVF outcome in such women, but many of these interventions are not evidence-based [5,6]. As a result, there is considerable variation in the approach to investigations and management of recurrent IVF failure [7].

One of the common investigations proposed after recurrent IVF failure is outpatient hysteroscopy (OH). OH is a well-tolerated minimally-invasive procedure, which allows reliable visual assessment of the cervical canal and uterine cavity and provides the opportunity to perform therapy in the same setting [5,8-11]. Intra-uterine pathologies have been shown to be present in 25% of infertile patients [11]. Thus, routine OH prior to IVF has been suggested by a number of investigators to ensure normality of the uterine cavity before embryo transfer [12-19] although this proposition has not been tested in a randomised setting.

In order to capture all existing evidence on whether OH could improve the outcome of the subsequent IVF cycle in patients who have experienced recurrent IVF implantation failure, we conducted a systematic review and meta-analysis [20]. We identified two randomized studies [21,22] which examined the impact of OH on the outcome of the subsequent IVF cycle after two or more failed IVF attempts. Both studies included patients with normal uterine cavity on hysterosalpingography. The researchers in both studies performed the hysteroscopy as an outpatient procedure, used a 5-mm hysteroscope with a 30° view, reported no complications and discharged patients within one hour after the procedure. Both studies showed a statistically significant improvement in the clinical pregnancy rate in the group who had OH (pooled RR = 1.57, 95%CI 1.29-1.92, p < 0.00001). The number needed to treat with OH in order to achieve an additional clinical pregnancy in the study populations was 7 (95%CI 5-12). The miscarriage rate was not statistically different between the OH and control groups [20].

However, there were deficiencies in the quality of these 2 randomised studies. For example, one study [22] did not report adequate concealment of allocation. Lack of concealment can exaggerate effect size by up to 41% [23]. In addition, only one of the studies [22] reported live birth rate per IVF cycle started. Furthermore, both studies were single-centre randomised studies, leading to limited generalisability of their findings. A well designed, adequately powered, allocation concealed multi-centre trial is therefore needed to address this research question.

## Methods and design

### Objective

In the proposed trial we will evaluate whether performing an OH prior to starting an IVF cycle improves the likelihood of achieving a live birth after the IVF cycle in women who have experienced two to four IVF implantation failures.

### Design

A prospective, allocation concealed, single blind, multi-centre randomised trial with health economic evaluation.

### Outcomes

The primary outcome is live birth rate (beyond 24 weeks gestation) per IVF cycle started. The secondary outcomes are embryo implantation rate, pregnancy rate per IVF cycle started, clinical pregnancy rate per IVF cycle started and miscarriage rate per pregnancy achieved

### Inclusion criteria

Women undergoing an IVF (with or without intracytoplasmic sperm injection) treatment cycle who have had between two to four fresh IVF cycles ending in an embryo transfer but no implantation are eligible to participate in the trial. For the purpose of this study, implantation is defined as the presence of an intra-uterine gestational sac showing fetal cardiac pulsations 4 - 5 weeks after embryo transfer.

### Exclusion criteria

Women who had less than two or more than four failed fresh IVF cycles ending in an embryo transfer, women aged 37 years or more at the time of randomisation, women who have had a hysteroscopy within 2 months of randomisation, the presence of submucous or intramural uterine fibroids distorting the uterine cavity or untreated tubal hydrosalpinges and a body mass index (BMI) above 35 (36 or higher).

The rationale for not including women aged 37 years and older is that the primary factor in IVF failure in this age group is related to embryo quality, which is unlikely to be corrected by uterine or endometrial manipulation. Submucous and intramural uterine fibroids distorting the uterine cavity and untreated tubal hydrosalpinges are known causes of IVF cycle failure and require specific treatment aimed at reversing their detrimental effects. Patients with a history of fibroids removal or treatment of tubal hydrosalpinges will not be excluded.

### Randomisation

Third party, distant, internet-based randomisation will be used to ensure randomisation and allocation concealment. Participants will be randomised online via a secure internet facility in a 1:1 ratio through a third party independent Integrated Trial Management System (MedSciNet Clinical Trial Framework) that has been designed, developed and delivered according to ISI9001-2000 standards and compliant with FDA CFR21:11 requirements. A "minimisation" procedure using a computer-based algorithm will be used to avoid chance imbalances in important stratification variables, such as age, BMI, number of previous failed IVF cycles and basal FSH level (as dichotomous variables). The randomised allocation will not be given until all eligibility and stratification data have been given.

### Blinding

The trial will be single-blinded, where the embryologists involved in the embryo transfer procedure are blinded to group allocation, thus minimising performance bias. Measurement bias in unlikely to interfere with the trial analysis and result due to the objective nature of the primary outcome (live birth).

### Methods

**Figure 1:**
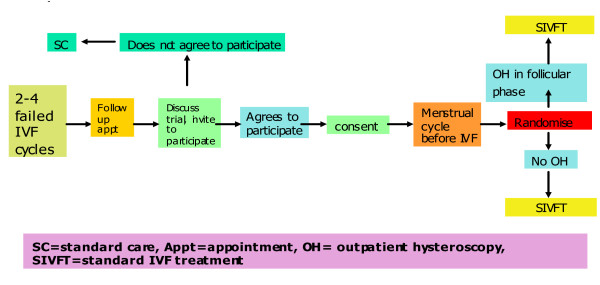
**Participants flow in the study**.

The flow chart in figure 1 describes the participant flow through the trial. Women with a history of two to four IVF implantation failures, who fulfil the inclusion and exclusion criteria, are offered information about the Trophy trial, and invited to participate.

If the woman agrees to participate in the trial, further information and instructions relating to the trial are given [see Additional file 1], and she is requested to sign the trial consent form [see Additional file 2] by a clinician. Contact and baseline data are collected in the electronic Integrated Trial Management System (ITMS) [see Additional file 3]. Each woman will be randomised to either the intervention or the control group.

All women are given instructions to notify the clinic nurse by telephone at the start of their menstrual cycle to inform her of their intention to start treatment in that cycle. When the nurse receives the call from the woman, the nurse will randomise the patient, and if the woman is allocated to the OH group, will book an OH within 14 days of the beginning of menstruation. Women randomised to OH arm will attend the respective Unit for OH within 14 days of the beginning of menstruation. They will then be expected to start the IVF process according to a standard controlled ovarian stimulation protocol [24]. Women randomised to the control group will start the IVF process according to a standard controlled ovarian stimulation protocol without OH.

### Trial intervention

#### Outpatient hysteroscopy (OH)

The OH procedure will be performed in the first two weeks of the menstrual cycle by named surgeons in each of the participating centres (Box 1):

##### Box 1: Hysteroscopy Equipment

*Hysteroscope*

▪ Rigid hysteroscope (continuous flow; 30° forward-oblique view) assembled in an examination sheath with an atraumatic tip giving a total instrument outer diameter of up to 3.2 mm (often called "micro-hysteroscopy") [25]. The telescope can be used in a single- or double-flow mode with an operative channel for 5 French instruments for minor operative procedures, thus increasing the total instrument diameter up to 5.0 mm. The operative 5 French instruments are either mechanical (such as crocodile forceps, biopsy forceps and sharp and blunt scissors) or electrical (such as bipolar needle or coagulation probe)

*Distension media*

▪ An isotonic solution (0.9% Normal saline or Ringer lactate) administrated via a pressure-controlled pump or simple pressure cuff system is used. The pressure is preset at between 80-120 mmHg with the aim to use the lowest pressure required to distend the uterine cavity adequately.

*Pre-medication*

▪ No routine pre-operative analgesics, antibiotics, sedatives or cervical preparation is used. A pelvic ultrasound scan should be performed before the procedure is started. All women must have a negative pregnancy test prior to commencing the procedure.

*Procedure*

▪ Before the hysteroscopy is performed, the woman is fully counselled by the clinician and requested to sign a consent form.

▪ The woman is positioned in the dorso-lithotomy (semi-recumbent) position using comfortable leg rests.

▪ The perineum should be at the edge of the couch with the coccyx and sacrum well supported by the table.

▪ Either the 'touch' (Cuscoe's speculum) or 'no touch' (vaginoscopy) techniques is acceptable.

◦ Antiseptic preparation of the ectocervix is at the discretion of the operator (e.g. chlorhexidine)

▪ Entry into the uterine cavity is under direct vision using gentle manipulation to minimise trauma to the cervical canal.

◦ The use of minimal cervical dilatation is to be restricted to cases of cervical stenosis. A tenaculum can be applied to the anterior lip of the cervix to help straighten the cervical canal.

▪ Local cervical anaesthesia (direct cervical or para-cervical infiltration) using short acting local anaesthetic (lidocaine or mepivicaine) may be administered at the discretion of the operator to facilitate a minor degree of cervical dilatation and after obtaining the woman's consent.

▪ On entering the uterine cavity a systematic inspection should be conducted to include

◦ Panoramic inspection of the uterine cavity at the level of the uterine isthmus

◦ Inspection under higher magnification of the uterine cornua, tubal ostia, uterine fundus, lateral walls, anterior and posterior uterine walls.

◦ Information to be recorded must include (See Additional file 3):

▪ The appearance of the endometrium.

▪ Shape of the uterine cavity [regular vs. irregular contour; normal vs. enlarged vs. tubular (restricted) size]

▪ Presence and location of structural anomalies (polyps, fibroids, adhesions, congenital anomalies)

▪ The entire intra-cervical canal should be inspected whilst slowly withdrawing the hysteroscope.

▪ Uterine dimensions estimated and recorded to include

◦ Cervical canal length (cm)

▪ Cervical canal length should be obtained by visualising the internal cervical os, with the distal tip of the hysteroscope at this level and the operator's finger pressed against the shaft of the telescope to mark the level of the external cervical os.

◦ Uterine sound length (cm)

◦ Uterine cavity length [uterine sound length - cervical canal length (cm)]

◦ If uterine polyps, small submucous fibroids (≤ 0.5 cm diameter) or filmy adhesions are identified, simultaneous mechanical hysteroscopic treatment should be attempted and the feasibility and outcome of such procedures recorded.

*Post-procedure*

▪ Patients should be observed for a minimum period of 30 minutes in a sitting recovery area before being allowed to leave the clinic. Simple analgesics (e.g. paracetamol or diclofenac) may be administered if required.

Clear and accurate surgical data will be routinely collected on each patient recruited via a specific 'hysteroscopy findings chart' [see Additional file 3]. These data will be analysed for specific findings, and for rates of adverse events or treatment failures.

The first outcome assessment (pregnancy test) will be done approximately two weeks after egg collection. The second and third outcome assessments (clinical pregnancy and implantation) will be done via ultrasound scan performed between 6-7 weeks of gestation at the respective trial centres.

The primary outcome assessment (live birth beyond 24 weeks) is conducted after delivery to gather data on delivery outcomes. A member of the local research team will also check birth registers and in-patient records to track hospital admissions and pregnancy outcomes.

### Trial statistics

#### Number of participants

Sample size calculation was based on the observed IVF cycle outcome and differences in outcome from existing literature as well as on the judgement on what constitutes as a clinically Minimally Important Difference (MID). The MID was judged to be increasing the live birth rate per cycle started by 10% (from 25% to 35%). For this difference of 10% increase in the live birth rate, and for a power of over 80% and a double sided alpha error of 5%, 720 women in total will need to be recruited (360 in each arm of the trial). In order to allow for a "worst case scenario" dropout rate of 5%, we intend to recruit 758 women in total (379 in each arm of the trial). The base rate of 25% was based conservatively on the control arms of the studies included in the published systematic review [20] and the MID of 10% was defined following consultations with fertility practitioners. This difference is much smaller than has been suggested by existing literature [22], which has reported an overall 75% relative improvement in IVF cycle outcome. Hence, assuming a conservative actual absolute difference of 15% (25% to 40%) in live birth rate beyond 24 weeks, 758 participants will provide a power of 96% at a double sided alpha error of 1%. Based on a comprehensive audit data covering over 4,000 IVF cycles, approximately 40% of patients seen in participating centres will be eligible for inclusion in the study. Assuming a modest 50% recruitment rate, the target of 758 patients could be randomised in 24 months. This recruitment rate is realistic and easily achievable given that there are no similar trials ongoing in participating centres, and IVF patients are generally motivated to participate in research [26].

#### Statistical analysis

The analysis will be by intention to treat, and will be carried out in the following four steps:

##### Step 1: Summarising trial data

Baseline and outcome data will be summarised separately. For continuous variables, we will examine the distribution of the observations, and if normally distributed we will summarise them as means with standard deviations (SD). If they are non-normally distributed, then medians and inter-quartile ranges (IRQs) will be reported. Appropriate transformation will also be considered. For dichotomous data (e.g. pregnancy, implantation, clinical pregnancy and live birth), we will provide proportions (or percentages). In addition to baseline and outcome data, we will also summarise the recruitment numbers, those participants lost to follow-up, protocol violations and other relevant data. We will prepare a CONSORT flow diagram to display these results.

##### Step 2: Inter-group comparisons

The statistical procedures for comparisons will depend on the nature of the data: for example, for dichotomous outcomes, we will use Fisher's Exact Test or Chi-square as appropriate, and for continuous outcomes we will use t-test if the observations in each trial arm are normally distributed; if non-normally distributed, then Mann-Whitney-U test will be employed.

We appreciate that multitudes of comparisons can suffer from Type I (false-positive) error, and will therefore determine the success or failure of the study in terms of the single primary endpoint on which the power calculations are based: the live birth rate. Although p-values will be reported, the focus will be on providing 95% confidence intervals around point estimates as these are more useful in interpreting the findings of the trial.

##### Step 3: Sub-group analysis

We will give emphasis to analysis within planned (a priori) sub-groups (namely normal versus abnormal hysteroscopic findings). However, we are aware sub-group analysis is limited by statistical power and can suffer from false positive (due to multiplicity of comparisons) and false negative (due to reduced sample sizes) results, and will place limited importance on sub-group analysis findings in relation to the overall (global) findings. We will use post-hoc sub-group analysis only for the purpose of hypothesis generation.

##### Step 4: Adjustments and sensitivity analyses

If randomisation fails to achieve balanced groups, then we will perform secondary analyses in which we will adjust for unbalanced prognostic factors using procedures such as logistic regression. If the primary unadjusted analysis and secondary adjusted analysis are at discordance, then we will give greater weighting to the primary analysis in the interpretation of trial findings. For issues such as losses to follow-up, missing data and protocol violations, we will attempt sensitivity analyses to explore the effect of these factors on the trial findings. As a secondary analysis, we will adjust for missing data using imputation techniques to explore the effects of such imputations on the trial findings.

### Interim Analysis and Data and Safety Monitoring

The trial will commission an independent Data Monitoring Committee (DMC). The DMC will have independent members with clinical and statistical background, who have no conflict of interest relating to the two trial arms and have no involvement in running any part of the trial. The DMC will be responsible for Data and Safety Monitoring.

During the trial, the DMC will perform interim analysis and review unblinded outcome data for safety and efficacy, initially after primary outcome data are available for 35% of the trial participants, and subsequently at six monthly intervals.

The DMC will review unblinded outcome data for safety and efficacy following interim analyses at intervals stated above applying the following Statistical Warning Rule, which is: (i) Doubling of the clinical pregnancy rate in one of the two groups compared to the other, with a statistical significance of P ≤ 0.001 on two consecutive interim analysis, (ii) Overwhelming evidence regarding the relative safety of the two trial arms, sufficient to make continuing randomisation unethical. The DMC will advice the Trial Steering Committee of any clear evidence that one approach is preferable or if there is an unacceptable level of serious adverse events.

### Withdrawal of Subjects

There will be no replacement of trial subjects who have withdrawn from the study, and as the analysis is by Intention-To-Treat (ITT), if outcome data are available for withdrawn study participants, they will be analysed according to the group to which they were randomised.

### Ethics and Confidentiality

This trial will be conducted according to the Principles of Good Clinical Practice as defined in the Medicines for Human Use (Clinical Trials) Amended Regulations 2006, the Research Governance Framework for Health & Social Care 2005 and the Data Protection Act. The trial has already received a favourable ethical opinion from a Regional Ethics Committee (REC).

Patient notes containing their personal and treatment details will be kept within the respective Units according to the statutory requirements of the HFE Act 1990 and the strict confidentiality that it requires. Notes from patients who have achieved a pregnancy will be kept or archived for 50 years. Patients will need to give their written consent before any of their treatment details or personal information is passed to their General Practitioners or any other persons who are not covered by an HFEA licence.

## Abbreviations

DMC: Data Monitoring Committee; HFEA: Human Fertilisation and Embryology Act; IRQ: Inter-quartile range; ISRCTN: International Standardised Randomised Controlled Trial Number; IU: International Unit; IVF: In vitro fertilisation; MID: Minimally Important Difference; OH: Outpatient Hysteroscopy; REC: Regional Ethics Committee; SD: Standard Deviation

## Competing interests

The authors declare that they have no competing interests.

## Authors' contributions

All authors contributed to the conception, planning and writing up of the trial protocol and revised the final version of the manuscript.

## Supplementary Material

Additional file 1 **TROPHY Trial Participant Information Sheet**. Gives information regarding the study to eligible women. Click here for file

Additional file 2 **TROPHY Trial Informed Consent Form**. To be signed by eligible consenting women prior to randomisation. Click here for file

Additional file 3 **Electronic Data Capture Form**. To record relevant study information for individual participants. Click here for file
