# Supplementary material for: A multi-centre randomised controlled study of pre-IVF outpatient hysteroscopy in women with recurrent IVF implantation failure: Trial of Outpatient Hysteroscopy - [TROPHY] in IVF
Source: Reprod Health. 2009 Dec 3;6:20. doi: 10.1186/1742-4755-6-20 (PMC2795733; doi:10.1186/1742-4755-6-20)
Supplement: Additional file 1 — TROPHY Trial Participant Information Sheet. Gives information regarding the study to eligible women. [file 1742-4755-6-20-S1.DOC]

# TO BE INSERTED ON LOCAL HOSPITAL PAPER

**Trophy**

**Trial of Outpatient Hysteroscopy (TROPHY)**

**in IVF**

**PARTICIPANT INFORMATION SHEET**

**LOCAL HOSPITAL TROPHY STUDY STAFF CONTACT DETAILS**

GYNAECOLOGIST NAME:………….……………………………………

GYNAECOLOGIST TEL: ………………….………………………………

RESEARCH NURSE NAME: ………………………………………………

# RESEARCH NURSE TEL: …………………………………………………

# Trial of Out Patient Hysteroscopy (TROPHY) in IVF

PARTICIPANT INFORMATION SHEET

You are invited to take part in a research study to find out if having a camera examination of the womb cavity (hysteroscopy) in the out-patient clinic before starting an IVF cycle increases the success rate of the IVF cycle. This study is called “Trophy” and compares out-patient hysteroscopy followed by IVF treatment with IVF treatment alone. The study is entirely voluntary – you do not have to take part, nor give a reason why, if you decide not to. Before you make your decision, it is important for you to understand why the research is being done and what it will involve. Please take time to read the following information carefully. You may want to talk to others about the study before taking part. If there is anything that is not clear, or you would like more information, you should ask your doctor or nurse for further advice.

- Part 1 of this information sheet tells you the purpose of this study and what will happen to you if you take part.
- Part 2 of this information sheet gives you more detailed information about the conduct of the study.

Ask us if there is anything that is not clear or if you would like more information. Take time to decide whether or not you wish to take part.

Part 1

What is the purpose of the study?

It is necessary during IVF treatment to have a normal womb cavity. Abnormalities of the womb cavity could lower of the chance of an IVF cycle being successful. These abnormalities could be:

1. A fibroid (growth of muscle cells in the womb which is not cancer)
2. A polyp (fleshy over growth of the tissue lining the womb cavity which is not cancer)
3. Thickening of the lining of the womb (the endometrium)
4. Adhesions (fibrous tissue in the cavity that could develop after womb infection or surgery)

Out-patient Hysteroscopy (internal camera examination of the womb cavity using a fine telescope) is a simple well-tolerated procedure which is done in the out-patient clinic setting while you are awake to diagnose and possibly treat abnormalities within the womb cavity. It is commonly performed by clinicians to evaluate the womb cavity. After two or more unsuccessful IVF treatment cycles, about three quarters of women who have hysteroscopy will have a normal womb cavity, whereas one fourth may have an abnormality. However, currently there is no reliable evidence to say whether performing out-patient hysteroscopy before an IVF treatment cycle improves the cycle outcome.

The purpose of this study is to find out if performing out-patient hysteroscopy before an IVF treatment cycle could improve the success rate of the treatment cycle in women who have had two or more unsuccessful IVF cycles.

**How will the out-patient hysteroscopy be performed?**

An out-patient hysteroscopy is a well-tolerated internal examination which uses a fine telescope, called a “hysteroscope”, to examine the lining and shape of the womb cavity and takes 5 minutes on average to complete. It is performed in the out-patient clinic, which means you will come into the hospital for the procedure and leave 1-2 hours later. It also means you can eat and drink as normal and take your usual medicine.

- Before the procedure your doctor or nurse will fully explain the procedure to you and ask you when your last period was, if you have any allergies and whether you have had any reactions to any drugs or tests in the past. Please do not hesitate to ask any questions about the procedure if you are uncertain. Please be reassured that the procedure will be immediately discontinued on your request at any stage.
- You will then be asked to sign the consent form.
- You will be awake during the procedure.
- You will be asked to lie on a couch with a nurse by your side
- An instrument, called a speculum and similar to the instrument used during a cervical smear test, is placed within the vagina to help the doctor see your neck of the womb (the cervix)
- The hysteroscope (a small fibre-optic telescope) is passed through the cervix in order to look at the inside of the womb.
- Sterile fluid is run through the telescope into the womb to expand it in order to help the doctor see the lining of the womb.
- After the lining of the womb has been examined, the doctor may introduce a small instrument through the telescope to remove any polyp, fibroid or scar tissue. Any tissue removed is sent to the laboratory for detailed examination.
- The procedure takes about 5 minutes on average. Please do not hesitate to tell the doctor or nurse if you feel any discomfort and we will give you pain relief for this.

**What happens after the procedure? And how will I feel?**

You will need to rest and have a cup of tea in comfortable surroundings in the hospital for about 15-30 minutes after the procedure. You might like to arrange for someone else to come to the appointment with you. You should be able to return to normal activities later that day or the next day. You can shower the day of the procedure.

You might feel some crampy discomfort, which is similar to a period pain and settle shortly after the treatment is completed. You can take pain relief such as paracetamol. If you find the pain is hard to control, please contact your GP or the ACU emergency telephone number on 07768 392313 for advice. A minority of women may feel a little faint following the procedure requiring them to lie down for a few minutes until the sensation passes. Light vaginal spotting or fresh blood loss is not uncommon, but should settle within a few hours of the procedure.

**What are the risks associated with an out-patient hysteroscopy?**

There are risks associated with any procedure. Out-patient hysteroscopy is a safe procedure and side effects occur in less than one in ten women. Your doctor or nurse will explain these risks to you before you sign the consent form.

The most common risks associated with this procedure are:

- Short lasting cramping discomfort in the lower abdomen like a “period pain” (occurs in about 1 in 20 women)
- Vaso-vagal reactions - a short episode of fainting associated with feeling cold and sweaty, which are usually self-limiting but may rarely require medical intervention in the form of an intravenous line and blood pressure support (occurs in about 1 in 100 women).
- Light vaginal spotting or fresh blood loss (occurs in about 1 in 100 women).
- Difficulty seeing the womb cavity (occurs in about 1 in 100 women).
- An infection requiring a short course of oral antibiotics (occurs in less than 1 in 100 women).
- Damage to the womb by making a hole in the wall of the womb called perforation (this is extremely rare and normally heals naturally).

**What should you look out for after the procedure?**

- *Vaginal bleeding*
  You might have light vaginal bleeding for up to seven days following the procedure. Please contact us if you experience heavy bleeding or pass any blood clots
- *Vaginal discharge*
  It is normal to have some vaginal discharge for up to seven days after the procedure. Please contact us if this discharge becomes offensive smelling as this might be a sign of an infection.
- *Tummy pain*
  You may experience cramping discomfort in the lower abdomen for few minutes after the procedure. Please contact us If you find the pain is hard to control with paracetamol.
- *High temperature* (38°C or above)
  Please contact us if this happens as it may be a sign of an infection.

**When will you get the results of the hysteroscopy?**

Before you go home, the doctor or nurse will talk to you about the results of the procedure and whether further treatment is needed. Information about your procedure will be sent by post to your GP and a photocopy of this letter will be sent to you.

**Why have I been invited?**

You have been given this study information leaflet as we have identified from your notes that you fit the criteria (in terms of outcome of previous IVF treatment cycles), which would make you eligible to take part. We aim to recruit 558 women to take part in the study over a period of 2 years.

**Do I have to take part?**

No. It is up to you to decide whether or not to take part. If you wish to take part, you will be given this information sheet to keep and will be asked to sign a consent form. You are still free to withdraw your consent at any time and without giving a reason. A decision to withdraw at any time, or a decision not to take part, will not affect the standard of medical care you receive in any way.

**What will happen to me if I take part?**

All participants will be randomly allocated by the central study office to either having the out-patient hysteroscopy or not. You will only participate in one of the two treatment regimens. After you have been allocated to a treatment regimen, the doctors and nurses will tell you exactly what you are to do in terms of the procedure and the subsequent IVF cycle. Depending on which group you are randomly allocated to, you will either have an out-patient hysteroscopy before you start your IVF cycle or you will start your IVF treatment without a hysteroscopy. If you are allocated to the out-patient hysteroscopy group, you will be asked to come to the out-patient IVF clinic after your period has finished to have the hysteroscopy. You will not have to make any extra-special trips back to hospital.

**Expenses and payments:**

The hysteroscopy will be provided to you free of charge as it is a study procedure. However, all IVF costs (the IVF procedure itself, hormone injections, the HCG injection and the progesterone pessaries) will need to be paid for you (either by your Primary Care Trust or by yourselves).

**What are the other possible disadvantages and risks of taking part?**

There are no disadvantages or risks of taking part in the study. Taking part in the research will not involve any extra tests or additional visits to the hospital except the one to have the procedure.

**What are the possible benefits of taking part?**

We cannot promise the study will help you personally in your next IVF treatment cycle but the information we will get from the study will help improve the future treatment of women who have experienced two or more failed IVF treatment cycle.

**What if there is a problem?**

If you have a complaint about the way you have been dealt with during the study or any other matter, you can lodge a complaint. There is more detailed information in Part 2 of this leaflet.

**Will my taking part in this study be kept confidential?**

Yes. The study will follow ethical and legal practice and all the information about your participation in this study will be kept confidential. Details about this are included in Part 2 of this leaflet.

**Contact Details:**

You should contact Mr El-Toukhy or Mr Khalaf at the Assisted Conception Unit on 0207 188 2300 or the address above for any further information or questions about this study. If you need to speak to a doctor outside routine working hours in case of an emergency, you should ring the Unit on 0207 188 2300 and you will be given the number for the on-call doctor from the Unit.

**This completes part 1 of the information sheet.**

**If the information in Part 1 has interested you and you are considering participating, please read the additional information in Part 2 before making any decision.**

**Part 2**

**What if relevant new information becomes available?**

If this happens, Mr El-Toukhy will tell you about it and discuss with you whether you want to or should continue in the study. If you decide not to carry on, Mr El-Toukhy will make arrangements for your care to continue. If you decide to continue in the study you will be asked to sign an updated consent form.

**What will happen if I do not want to carry on with the study?**

It is entirely up to you whether or not you wish to take part. If you decide to take part and then change your mind you are free to withdraw at any time without giving a reason (although it would be useful to know why) and your treatment/care will not be affected in any way.

**What if there is a problem?**

**Complaints**

You have the same legal rights whether or not you take part in this study. Any complaint about the way you have been dealt with during the study will be addressed. If you are not satisfied with any aspect of the way you have been approached or treated during the course of this study, you should ask to speak to Mr El-Toukhy or Mr Khalaf who will try their best to answer your questions (contact number 0207 188 2300 or at the above address). If you remain unhappy and wish to complain formally, you can do this through the NHS complaints procedure. You can contact the Patients Advisory and Liaison Service (PALS) at Guy’s and St Thomas’ NHS Foundation Trust or you can write to Mr Ron Kerr, Chief Executive, Guy’s and St Thomas’ NHS Foundation trust, Guy’s Hospital, Great Maze Pond, London, SE1 9RT.

**Harm**

Should you be harmed in any way by this study, unfortunately we cannot provide special compensation arrangements. If you are harmed and this is due to someone’s negligence then you may have grounds for a legal action for compensation against Kings College London and Guys and St Thomas’ NHS Foundation Trust, but you may have to pay your legal costs. The normal NHS complaints mechanisms will still be available to you.

**Will my taking part in this study be kept confidential?**

Yes. All information collected from you for the purposes of this study will be kept strictly confidential in the same way as your medical records. Any information used outside the hospital will have any identifying details removed so that your data remains completely anonymous. All information will be held securely and in strict confidence. You will not be identified in any publication of results from this study. We will inform your GP of your participation in the trial if you agree. Occasionally, inspections of clinical study data are undertaken to ensure that, for example, all participants have given consent to take part. But apart from this, only study organisers will have access to the data.

**What will happen if I lose capacity to consent during the study?**

If you lose capacity to give consent during the study, any data which is not identifiable may be retained. Any identifiable data would be anonymised or disposed of.

**Involvement of your General Practitioner (GP)**

We will inform your GP of your participation in the trial if you agree.

**What will happen to the results of the research study?**

When the results of the Trophy study are known, they will be published in medical journal(s). We will also make the information available on our website for the general public. The results will influence the way women with more than one failed IVF cycle are treated in the future.

**Who is organising the research?**

The study is being organised by the Assisted Conception Unit, Guys and St Thomas’ NHS Foundation Trust.

**Do you have any further questions?**

Having read this leaflet, it is hoped that you will choose to take part in the Trophy trial. If you have any questions about the study now or later feel free to ask your gynaecologist or clinic nurse. Please take the time before your appointment to decide whether you wish to take part in the Trophy trial. You may like to discuss your decision with friends or relatives.

The UK Clinical Research Collaboration has produced a guide entitled, ‘Understanding Clinical Trials’. This can be down loaded from their website: [www.ukcrn.org.uk](http://www.ukcrn.org.uk/) and maybe useful if you require general information about research. If you require specific information about the research project please contact your doctor or nurse.

**You will be given a copy of the information sheet and a signed consent form to keep.**

**Thank you for taking time to read this sheet and for considering taking part in the study.**
