# Supplementary material for: A multi-centre randomised controlled study of pre-IVF outpatient hysteroscopy in women with recurrent IVF implantation failure: Trial of Outpatient Hysteroscopy - [TROPHY] in IVF
Source: Reprod Health. 2009 Dec 3;6:20. doi: 10.1186/1742-4755-6-20 (PMC2795733; doi:10.1186/1742-4755-6-20)
Supplement: Additional file 2 — TROPHY Trial Informed Consent Form. To be signed by eligible consenting women prior to randomisation. [file 1742-4755-6-20-S2.DOC]

**Centre Number:**  0102

**Study Number:**

**Name of Researchers:**

**Patient Identification Number for this trial:**

**CONSENT FORM**

**Title of Project: Trial of Out-Patient Hysteroscopy in IVF (Trophy Trial)**

1. I confirm that I have read and understand the information sheet for this study:

***(“***Trial of Out-Patient Hysteroscopy in IVF***” Version 4, 12/03/2009)***

I have had the opportunity to consider the information, ask questions and have these answered satisfactorily.

1. I understand that my participation is voluntary and that I am free to withdraw at any time, without giving any reason and without my medical care or legal rights being affected.
2. I give permission for responsible individuals from the Guy’s Assisted Conception Unit to have access to my medical notes (where it is relevant to my taking part in the research).
3. I agree to my GP being informed of my participation in the study.
4. I agree to take part in the above study.

Name of Patient Date Signature

Name of Person taking consent Date Signature

(if different from researcher)

Researcher Date Signature

| When completed: |  |
| --- | --- |
| - 1 form for participant |  |
| - 1 for researcher site file |  |
| - 1 (original) to be kept in medical notes. |  |
