# Supplementary material for: A multi-centre randomised controlled study of pre-IVF outpatient hysteroscopy in women with recurrent IVF implantation failure: Trial of Outpatient Hysteroscopy - [TROPHY] in IVF
Source: Reprod Health. 2009 Dec 3;6:20. doi: 10.1186/1742-4755-6-20 (PMC2795733; doi:10.1186/1742-4755-6-20)
Supplement: Additional file 3 — Electronic Data Capture Form. To record relevant study information for individual participants. [file 1742-4755-6-20-S3.DOC]

**Registration**

**Trophy EDC** (Electronic Data Capture) **and TMF** (Trial Management Framework)

Tarek El-Toukhy Version 14 Creation date: 26/08/2008 Updated: 12/05/2009

Local hospital number

NHS number (or an equivalent) [ideally we need two unique identifiers; a national identifier (like the NHS number) may allow tracing of those who may have left the unit where they received treatment)

Name* text

Date of birth* date

*[CTF to display Age at registration numeric (years and days)]*

Address* text

Telephone* numeric

Mobile* numeric

Email* text

Telephone for next of kin* numeric

Centre drop-down (8 centres)

*Registered by:* drop-down

* *These data will be collected on a local database and held securely to adhere with Data Protection Act and Caldicott requirements. Local hospital number will allow linking of EDC/TMF to data held locally.*

**Inclusion/ Exclusion Criteria**

***Inclusion criteria***

Responses below must all be “**Yes”** to be eligible.

Patient willing to participate in trial. binary radio button

No clinical pregnancy after last 2 to 4 fresh IVF/ICSI cycles binary radio button

reaching embryo transfer

Age < 37 (36 years and 364 days or less) binary radio button

No hysteroscopy in the previous 2 months binary radio button

***Exclusion criteria***

Responses below must all be “**No” or “NA” (not applicable)** to be eligible.

BMI 35 or more binary radio button

Submucosal or intramural fibroids distorting cavity binary radio button

Hydrosalpinx not removed, disconnected or drained binary radio button

*If inclusion/exclusion criteria met, provide (verbal and written)* ***information and****, obtain* ***written******consent.*** *Ask patient to call the research nurse on* ***day 1-3*** *of period to randomise and order medication for IVF cycle****.***

If an eligible patient declines to participate, please record:

Reason for not participating drop down menu (does not wish to participate in a trial, does not wish to have a hysteroscopy, insists on having a hysteroscopy, other (free text)

Did she have a hysteroscopy? binary radio button

Outcome of treatment cycle as study patients (see later)

**Medical Data**

Age Auto calculation by TMF

Height

Weight

*BMI (for autocalculation by CTF)*

Previous pregnancies (any) drop down (none, 1, 2, 3 or more)

Previous IVF clinical pregnancies (any) drop down (none,1, 2, 3 or more)

Previous live birth (beyond 24 completed weeks) drop down ( none,1, 2, 3 or more)

Fibroids drop down of 4 options (none, intramural non-cavity distorting, subserosal or both) If any answer other than none, please add number (1-10) and diameters (1-900mm in 3 planes i.e. axbxc mm) for each fibroid separately.

Gynaecological surgery drop down (myomectomy, Caesarean section, Loop excision, other)

Medical history drop down (Diabetes, hypertension, asthma, heart disease, thyroid dysfunction, autoimmune condition, free text for other)

Medications drop down (list as promise, with free text for other)

Current smoker binary radio button

Duration of infertility (years) numeric

Cause of infertility drop down option boxes

Tubal

Male

Endometriosis

Anovulation

Unexplained

Combined

Number of previous fresh IVF/ICSI cycles with no pregnancy drop down (2, 3, 4)

Number of previous frozen IVF/ICSI cycles with no pregnancy drop down (0, 1, 2, 3, 4, 5 or more)

Baseline investigations

Date of test date (can be before date of recruitment)

Serum FSH (day 2-5 of cycle) numeric (one decimal space, 1-99.9)

Serum Estradiol (day 2-5 of cycle) (non-obligatory) numeric (1-1000)

Antral follicle count (AFC any time in cycle) (non-obligatory) numeric (1-100)

Uterine ultrasound findings: (non-obligatory) binary button (normal, abnormal) if abnormal (free text for “describe)

Ultrasound uterine length: (non-obligatory) numeric (1-10 cm)

**Please add the option of “save draft” here before randomisation. Then we should have a button for “Randomisation”**

**Participants are randomised (with minimisation) when they call on day 1-3 of cycle. Computer allocates a unique participant number. Randomisation at this stage allows for minimisation for prognostic variables [Age <31, 31-36; BMI <30, 30-34; number of previous failed fresh cycles 2, 3-4; basal FSH level <10 or ≥10).**

| **Allocation**  Out-patient hysteroscopy No out-patient hysteroscopy |
| --- |

**Intervention:**

**Out-patient hysteroscopy performed (proceed to out-patient hysteroscopy)**

**No out-patient hysteroscopy (proceed to IVF details)**

**Out-patient hysteroscopy:**

Date of hysteroscopy

Operator drop down (list of maximum 4 operators per centre)

Grade drop down (consultant, senior trainee, senior nurse)

Visualisation binary radio button ( sufficient and insufficient) if insufficient, drop down for bleeding, pain, vasovagal reaction, technical failure, other free text more than one answer acceptable please) and then move to complications. if sufficient, proceed to details as below.

Details:

. Cervical canal drop down [a-normal or b-abnormal, (drop down menu for stenosed external os, stenosed internal os, obliterated canal, polyp, other free text more than one answer acceptable please)

. Uterine axis drop down (anteflexed, axial or retroflexed)

. Endometrial cavity drop down [a-**normal**, b-**abnormal** congenital malformation (if yes provide options:1- arcuate, 2-partial septum, 3-complete septum, 4-T-shaped cavity or 5-other free text to describe), submucous fibroid (if yes provide options of 1-Size: <1cm or ≥1cm, 2- Number: 1 or >1, 3- Grade: 0, 1 or 2 (help text: Grade 0 - pedunculated, Grade 1 - projecting more than 50% in the cavity, Grade 2 - projecting less than 50% in the cavity)), polyp >5mm, adhesions (if yes provide options of: type: filmy, fibrous, cavity obliterated <10%, 10-50% or >50%)]

. Endometrium drop dowm (a- normal or b- **subtle endometrial lesions** including mucosal elevation, if so give option of cystic or solid, reddish endometrium, (if so give options of hypervascularisation, strawberry pattern or diffuse polyposis), necrotic lesion, endometrial defect, single fine adhesion band, other +free text) more than one answer acceptable please

. Right tubal ostium drop down (a- seen, b- not seen c- other +free text, )

. Left tubal ostium drop down (a- seen, b- not seen c- other +free text,)

. Operative intervention (more than one answer acceptable please) drop down (a-none, b- immediate, c-scheduled) if b or c, then proceed to a new window with either minor or major intervention. Options for minor include a-cervical dilatation, b- one fine band of adhesion divided mechanically, c-polyp ≤15mm removed mechanically, d-fibroid ≤5mm resected mechanically, e-other please describe. Options for major include a- more than 1 polyp removed b-polyp >15mm removed, c-fibroid >5mm resected, d-division of more than one fine band of adhesion, e- endometrial resection, f- division of partial or complete septum g-thermal energy used, h-listed for another hysteroscopy) open a new window with same data entry options. First two question should be: type of hysteroscopy drop down (out-patient, day surgery, in-patient)

Anaesthetic given drop down (none, local anaesthetic, local anaesthetic and sedation, sedation only, general anaesthetic)

. Uterine length number (1.0-15.0 cm)

measurement made using drop down (ultrasound, uterine sound,, catheter, other free text)

1. Complications:
   Intra-operative drop down
    (none, pain, vasovagal fainting, bleeding, perforation, fluid overload, abandoned, other free text)

   Post-operative drop down
    (none, pain, vasovagal fainting, bleeding, infection, hospital admission, other free text)
2. Resource use:
   time of procedure numeric (minutes)
   time to discharge numeric (minutes)
   medication given binary button
   type of medication given drop down (sedation, oral analgesic, local anaesthetic, antibiotic, other free text)
   name of each medication used free text
   follow up appointment (hospital, GP or nurse) binary button

**IVF cycle:**

Has patient had uterine length measured binary radio button

during treatment cycle **(for the no out-patient hysteroscopy group only)**

If yes, measurement made using drop down (ultrasound, uterine sound,, uterine catheter, other free text)

IVF treatment protocol drop down:

GnRH agonist long

GnRH agonist short

GnRH antagonist

Other (free text)

Gonadotrophin type drop down

Gonal –F

Puregon

Menopur

Pergoveris

Merional

Other (Free text)

Dose of gonadotrophin for ovarian stimulation per day numeric (10-999)

No of days of gonadotrophin stimulation numeric (1-99)

Total dose of gonadotrophin used calculate (100-10,000) accept override to allow for dose changes during the stimulation days + please add this as help text to inform whoever is adding data

HCG type drop down:

Pregnyl

Ovitrelle

HCG dose numeric (1,000 – 99,000)

Did patient have an egg retrieval? binary button (if yes, proceed. If no, go to end of report)

Date of egg retrieval date (must be after LMP date)

Number of eggs retrieved numeric (0-99)
Method of fertilisation binary button (IVF or ICSI)
 If IVF, number of eggs inseminated. If ICSI, number of eggs injected (0-99)

Number of eggs fertilised numeric (0-99)

Did patient have an embryo transfer? binary button (if yes, proceed. If no, proceed to did patient have embryo freezing?, and if no, go to end of report, if yes, proceed to no of embryos cryopreserved, then go to end of report)

Number of embryos transferred numeric (1-9)

At least one top quality embryo* binary button

Day of embryo transfer numeric (1-6)

Embryo transfer procedure drop down (easy, difficult, stylet used, other free text) Please add as help text: Easy transfer is defined as: straight forward transfer requiring no additional intervention. Difficult: any other type of transfer.

Did patient have embryo freezing? binary button (if yes, proceed to next question)

No of embryos cryopreserved numeric

Day of freezing numeric (1-6)

**Urinary/serum pregnancy test binary radio button (positive / negative)**

**[if positive, proceed to below]**

**____________________________________________________________________________**

Outcome point 1 (ultrasound 6 – 8 weeks)

Date of scan

Days since egg retrieval

Calculate gestation in weeks+days (days since date of egg retrieval + 14 days)

Number of fetal sacs Drop down (0 – 3, more)

Expected date of delivery calculate (38 weeks after date of egg retrieval)

History of PV bleeding? Binary radio button

Any other comments free text

*[The following will need to be duplicated for each sac]*

Yolk sac seen Binary radio button

Fetal pole seen Binary radio button

Fetal heart seen Binary radio button

Any other comments free text

_____________________________________________________________________

Outcome point 2 (ultrasound 9 – 12 weeks) Please make this data non-obligatory

Date of scan

Days since egg retrieval

Calculate gestation in weeks+days (days since date of egg retrieval + 14 days (add this to help text))

Number of fetal sacs Drop down (0 – 3, more)

History of PV bleeding? Binary radio button

Any other comments free text

*[The following will need to be duplicated for each fetus]*

Yolk sac seen Binary radio button

Fetal pole seen Binary radio button

Fetal heart seen Binary radio button Any other comments text _____________________________________________________________________

Outcome set 3 (Pregnancy loss details if applicable)

Miscarriage binary radio button

Gestation of miscarriage numeric

Management of miscarriage drop down:

conservative

medical

surgical

Ectopic pregnancy binary radio button

Management of ectopic drop down:

conservative

medical

surgical

Outcome point 3 (Delivery and newborn outcome)

Number of foetuses/babies drop down (1 – 3, more)

*[The following will need to be duplicated for each fetus/baby]*

Date of birth date

Gestation at birth in weeks numeric

*The following options if < 24 weeks:*

Mode of delivery Vaginal/ Caesarean

Comments free text

*The following options if >/= 24 weeks:*

Mode of delivery Vaginal/ Abdominal

Born alive? binary radio button

Birth weight numeric

Comments free text

**End Report**

Did the patient complete the study binary radio button

If “no”, what was the reason? check boxes:

*adverse events (if yes, describe, free text)*

*lost to follow up*

*other (describe)*

Withdrawal date date

**Monitoring needs:**

Prognostic balance chart

Follow up alerts (2 week after EDD, send an e-mail alert to user, centre administrator and trial manager – so we need e-mail addresses of both)

Recruitment rates by centre (including non-recruitment for eligible patients)

Number of each complication by centre

Basic alerts

* a 4-cell embryo on day 2 or 7/8-cell embryo on day 3 with even blastomere size and no or less than 10% fragmentation by volume or an expanded blastocyst with prominent and compact ICM and many identical trophectoderm cells forming a continuous layer on day 5 or 6 after fertilisation (BFS and ACE Guidelines for Practice, 2008).
